# Effects of Alcohol Binge Drinking and Oleoylethanolamide Pretreatment in the Gut Microbiota

**DOI:** 10.3389/fcimb.2021.731910

**Published:** 2021-11-23

**Authors:** Alicia Rodríguez-González, Francesco Vitali, Marta Moya, Carlotta De Filippo, Maria Beatrice Passani, Laura Orio

**Affiliations:** ^1^ Laboratory of Psychobiology, Department of Psychobiology and Methods in Behavioral Science, Faculty of Psychology, Complutense University of Madrid, Madrid, Spain; ^2^ Institute of Agricultural Biology and Biotechnology (IBBA), National Research Council (CNR), Pisa, Italy; ^3^ Dipartimento di Scienze della Salute, Università di Firenze, Firenze, Italy; ^4^ Red de Trastornos Adictivos (RTA), Instituto de Salud Carlos III (ISCIII), Madrid, Spain

**Keywords:** fecal microbiota, alcohol, OEA, microbiome, substance abuse, binge, dysbiosis

## Abstract

**Introduction:**

Chronic alcohol consumption is known to cause gut dysbiosis (changes in microbiota composition and/or function, disruptive of the normal host–microbiota interactions). However, little is known about the changes that alcohol binge drinking induces in the gut microbiota. Here, we have tested the hypothesis that a protocol of alcohol binge drinking, known to induce neuroinflammation in previous studies, also promotes intestinal dysbiosis, and we explored how oleoylethanolamide (OEA, an acylethanolamide proven to counteract alcohol binge drinking-induced neuroinflammation) pretreatment modulates alcohol-induced dysbiosis.

**Methods:**

Alcohol binges were forced by gavage three times per day during 4 consecutive days; OEA pretreatment (intraperitoneal or intragastric) was administered before each alcohol gavage. Stool microbiota composition was assessed by next-generation 16S rRNA gene sequencing, prior and after the 4-day alcohol binge protocol.

**Results:**

Alcohol binge drinking reduced the richness of the gut microbiota and changed the microbial community, reducing *Lactobacillus* among other genera. Pretreatment with OEA in the alcohol-administered rats decreased the richness, evenness, and Shannon indices to a greater extent with respect to alcohol alone, also changing the community structure. Microbial interactions in the association network were further decreased following OEA administration in the alcohol group, with respect to the water administration. The synergistic interaction between alcohol binge and OEA was affected by the route of administration of OEA, since oral and i.p. administrations differently changed the community structure.

**Conclusion:**

Results suggest that alcohol binge drinking produces a clear dysbiosis in animals; we observed that the well-known protective actions of OEA in the context of alcohol abuse might not be related to OEA-induced changes in alcohol-induced dysbiosis. These are observational results, and thus, further research will be needed for a complete understanding of the biological significance of the observed changes.

## Highlights

- After an alcohol binge drinking protocol (T1) in rats, we observed reduced richness of the fecal microbiota, with a distinguishable bacterial community from T0 (no treatment) and from the T1 water group. Alcohol increased the levels of *Clostridium sensu stricto*, *Romboutsia*, *Turicibacter*, *Bifidobacterium*, *Parabacteroides*, and *Enterococcus* and decreased the levels of *Lactobacillus*, *Clostridium* XIVa, *Clostridium* XIVb, and *Oscillibacter*.
- OEA administration in the water group slightly changed the community structure with respect to the vehicle group but did not change the α-diversity. Microbial interactions in the association network were increased following OEA administration in the water group.
- Alcohol and OEA appeared to synergistically interact with the gut microbiota. Pretreatment with OEA in the alcohol-administered rats decreased the richness, evenness, and Shannon indices and changed the community structure to a greater extent in comparison with the alcohol without OEA group. Microbial interactions in the association network were further decreased following OEA administration in the alcohol group with respect to the water group.
- The route of OEA administration had a significant effect in the altered community structure induced by alcohol. Oral OEA reduced richness and i.p. OEA created a more fragmented association network, with more non-interconnected nodes, in comparison with vehicle administration in alcohol rats.

## Introduction

Alcohol abuse is a risk factor for many diseases and mortality worldwide. Alcohol binge drinking is a pattern of alcohol abuse defined as the consumption of more than four or five drinks in about 2 h for women and men, respectively, resulting in blood alcohol levels (BAL) ≥80 mg/dl (NIAAA, 2004). In 2015, in the National Survey on Drug Use and Health, it was shown that 26.9% of U.S. adults older than 18 years old reported binge drinking in the past 30 days (SAMHSA, 2017). Other reports indicate that 38 million of U.S. adults drink alcohol in this pattern four times per month, consuming eight drinks on average, and that half of the 88,000 deaths resulting from alcohol consumption are due to binge drinking (Kanny et al., 2015).

Alcohol binge drinking appears to promote alterations in the gut–brain axis. We have previously demonstrated that the binge drinking protocol used in this study induces gut barrier alterations, with consequences in peripheral inflammation and immune activation, neuroinflammation, and behavioral changes. On one hand, binge drinking induces colonic inflammation and a decrement of tight junction (TJ) protein expression in the colonic barrier, an indication of its loss of integrity. As a result, the cell wall component of Gram-negative bacteria, i.e., lipopolysaccharide (LPS), is found in the blood after alcohol binges, and interestingly, we also observed the passage of bacteria from the intestinal lumen to other organs, including the mesenteric lymph nodes (MLNs) and spleen. This pattern of alcohol binge drinking strongly activates the innate and adaptive immune systems and induces peripheral inflammation and corticosterone rise in plasma (Antón et al., 2017; Antón et al., 2018). On the other hand, it is well-known that binge drinking induces neuroinflammation in the frontal cortex, which has been related to anxiety and depressive-like behavior during early withdrawal (Antón et al., 2017).

The microbiota–gut–brain axis is a bidirectional communication between the microorganisms present in the gut and the brain that uses the immune system and neural and neuroendocrine pathways (Cryan and Dinan, 2012). Gut microbiota is composed of a plethora of microorganisms (predominantly bacteria, but also archaea, viruses, fungi, and protozoa among others) that live in different niches of our gastrointestinal tract, especially in the colon, in a symbiotic relationship with the host, cooperating in different functions such as digestion, immunity, inflammation, and intestinal barrier regulation (Wang and Wang, 2016). Additionally, it has been proposed that the gut microbiota participates in the regulation of brain function, mood, and behavior through modulation of the gut–brain axis (Cryan and Dinan, 2012).

Alterations in this axis have been well documented in several brain disorders and conditions (Sherwin et al., 2018), including alcohol use disorders (AUD) (Rodriguez-Gonzalez and Orio, 2020). It has been recently shown that AUD leads to gut dysbiosis in humans by altering the composition and/or function of the microbiota (Litwinowicz et al., 2019). In experimental models, alcohol generally increases bacteria from the phyla Proteobacteria and Firmicutes and decreases bacteria from the phylum Bacteroidetes (Engen et al., 2015). Alcohol-induced gut dysbiosis, together with the disrupted intestinal barrier and the LPS leakage to the systemic circulation, is associated with alcoholic liver disease, which is a leading cause of mortality worldwide (Hartmann et al., 2015; Meroni et al., 2019), and with behavioral abnormalities in alcohol-dependent subjects (Leclercq et al., 2014; Temko et al., 2017). Indeed, alcohol-induced alterations in gut microbiota may account for withdrawal symptoms such as anxiety, depression, craving, and reward-seeking behaviors (Hillemacher et al., 2018). However, alcohol consumption does not always cause gut dysbiosis, highlighting that there may be other factors involved (Mutlu et al., 2012; Timary et al., 2015) and that patterns of alcohol consumption must be taken into account. The actions of binge drinking on neuroinflammation are very well known, and we recently described binge drinking-induced dysfunction of the gut barrier that may influence cortical neuroinflammation (Antón et al., 2018; Orio, 2020; Rodríguez-González and Orio, 2020). Further research is needed to understand whether the gut–brain axis alterations induced by this pattern of alcohol consumption involve also changes in the gut microbiota composition and possible modulatory pharmacotherapies. In this sense, addressing the gut microbiota composition could offer a new therapeutic option to combat alcohol-induced neuroinflammation and toxicity.

Previous studies in Orio’s laboratory showed that pharmacological pretreatment with oleoylethanolamide (OEA) before each binge episode prevents the alcohol-induced harmful effects on the gut–brain axis. OEA showed potent anti-inflammatory, antioxidant, and neuroprotective actions by blockade of Toll-like receptors (TLR)-4 and its agonists in the frontal cortex of rats. For example, 1) OEA ameliorates LPS-induced neuroinflammation in the frontal cortex and modulates motivated behaviors such as anhedonia (Sayd et al., 2015) and 2) OEA reduces alcohol binge-induced neuroinflammation in the frontal cortex and depressive-like behavior during abstinence (Antón et al., 2017). In Antón et al. (2018), we showed that OEA is able to reduce the leakage of bacterial components such as LPS (a TLR-4 agonist) from the gut to the blood and even the passage of whole bacteria to the MLNs, since it prevents alcohol-induced gut inflammation and decreases tight junction (TJ) proteins of the colonic barrier (Antón et al., 2018). These findings provide an exciting evidence of the participation of OEA in the dysregulation of the gut–brain axis induced by alcohol. However, the role of OEA in the modulation of microbiota composition, as part of the mentioned microbiota–gut–brain axis, has not been studied yet in the context of alcohol abuse.

Independently, Passani’s and de Filippo’s laboratories studied the effect of OEA administration on microbiota profile in healthy mice, showing that OEA changes the gut microbiota composition, expanding the microbial diversity, increasing Bacteroidetes, and decreasing Firmicutes, which is very similar to a lean-like phenotype fed with a fiber-rich diet (Di Paola et al., 2018). Thus, in the present study, we joined the expertise of the three previously mentioned research groups, to ascertain the specific effects of OEA pretreatment in the gut microbiota profile in the context of alcohol binge drinking consumption.

## Materials and Methods

### Animals

Forty-two young adult male Wistar rats (Envigo^®^, Barcelona, Spain) 8–9 weeks old, weighing around 160–220 g, were used. Rats were housed in cages in groups of three to four rats with a reverse 12-h light/dark cycle under standard temperature and humidity at the SPF Animal Care Facility of Complutense University of Madrid. Animals were allowed free access to standard food (A04 SAFE, Scientific Animal Food and Engineering, Augy, France) and tap water. Rats were maintained and daily surveilled for 10 days under constant conditions before the experiments. All studies were designed in compliance with the ARRIVE guidelines (Kilkenny et al., 2010) and adhered to the guidelines of the Animal Welfare Committee of Complutense University of Madrid (reference: PROEX 420/15) according to European legislation (2010/63/UE). The study groups were as follows: experimental groups: T0 rats (before any treatment) and T1 groups (after treatments): water + vehicle group, water + oral/i.p. OEA group, and alcohol + oral/i.p. OEA group.

### Alcohol Binge Intoxications

Alcohol (30%, v/v) or vehicle (tap water) was intragastrically administered with specific cannulae (16G needle, Fisher Scientific, Waltham, MA, USA) at a maximum of three times/day during 4 consecutive days, following a standard paradigm of alcohol binge drinking (Obernier et al., 2002; Antón et al., 2017; Antón et al., 2018). The doses were titrated by blood alcohol levels (BAL) and by the observed behavioral signs related to alcohol intoxication (Antón et al., 2017; Antón et al., 2018). The average dose of alcohol per rat was 8.30 g/kg/day in this experiment.

The number of rats per group was initially higher in the alcohol-fed groups, since this protocol often induces mortality. In this experiment, the mortality was around 8%, similar to other studies (Obernier et al., 2002; Antón et al., 2017; Antón et al., 2018).

### Drugs

OEA was synthesized as described in Giuffrida et al. (2000). OEA i.p. was dissolved in 5% Tween 80 in saline (vehicle) and administered at 5 mg/kg i.p., 10 min before the alcohol binges, except for the loading initial dose that was 10 mg/kg. Control rats were injected with vehicle i.p. This protocol of OEA administration has been proven to reduce binge drinking-induced peripheral inflammation and neuroinflammation (Antón et al., 2017; Antón et al., 2018).

OEA orally administered was dissolved in 1% carboxymethylcellulose in saline and administered at 20 mg/kg i.g. (3 ml/kg) 20 min before each alcohol gavage with specific cannulae (16G needle, Fisher Scientific, Waltham, MA, USA). This dose of OEA oral administration has been previously shown to prevent the binge drinking-induced bacterial translocation from the intestinal lumen to other organs, such as the MLNs, as well as the passage of LPS to the systemic blood (Antón et al., 2018).

### BAL Determination

Blood samples were collected (20 µl) from the rat tail 2 h after the second alcohol administration every experimental day (at 3 p.m.). BAL were measured by enzymatic reaction using electrochemical detection with AM1 Alcohol Analyzer (Analox Instruments, London, UK). This protocol of alcohol binge drinking led to a sedation/ataxia behavior and relatively constant intoxicating BAL (average alcohol levels/day: 252.42 ± 165.58 mg/dl).

### Fecal Sample Collection and Bacterial Genomic DNA Extraction

Feces were collected in sterile conditions before treatments (T0) and at the end of the 4-day protocol, 3 h after the last alcohol/water administration (T1), and stored at −80°C until nucleic acid extraction. Bacterial genomic DNA was extracted using DNeasy PowerLyzer PowerSoil Kit (Qiagen^®^, Hilden, Germany) following the instruction of the manufacturer. DNA quality was examined by gel electrophoresis.

### 16S Ribosomal DNA Sequencing

For microbiota analysis, the V3–V4 hypervariable regions of the bacterial 16S rRNA gene were amplified using a specific primer set (341F: 5′-CCTACGGGNGGCWGCAG-3′ and 805r: 5′-GACTACNVGGGTWTCTAATCC-3′) (Takahashi et al., 2014). Sequencing was performed with Illumina MiSeq (Illumina, San Diego, CA, USA) with V3 chemistry (600 cycles) with a PE 2X300 protocol at the IGA Technology Services (Udine, Italy) following their internal protocol. Raw reads data are available at the European Nucleotide Archive (ENA) database under accession number PRJEB44965.

### Sequencing Data Processing and Statistical Analysis

Similar to previous reports (Butera et al., 2020; Tamburini et al., 2020), the obtained sequence libraries were pretreated by removing sequencing primer with CUTADAPT (Martin, 2011) and the low-quality ends at 5′ by using Sickle (Joshi and Fass, 2011) with a quality cutoff of 20. Pretreated sequences were then analyzed using MICCA v 1.7.2 (Albanese et al., 2015) to perform joining of forward and reverse reads and to perform amplicon sequence variance (ASV) picking using the UNOISE3 algorithm (Edgar, 2016). Taxonomy assignation to representative sequences of the identified ASVs was performed using RDP classifier and database v. 2.11 (Wang et al., 2007). Data were then analyzed using R software with a variety of dedicated packages. Phyloseq v.1.28.0 (McMurdie and Holmes, 2013) was used to import and handle ASV table and taxonomy files obtained through MICCA as well as to calculate PCoA ordination analysis (using the Bray–Curtis distance measure). Rarefaction to the smallest library size was used to normalize ASV counts prior to α-diversity analysis, which was performed using the microbiome package v 1.6.0 (Lahti et al., 2017). Other analyses were performed on the cumulative sum scaling (CSS transform, followed by log2 scaling) normalized ASV count table, as implemented in the metagenomeSeq version 1.26.3 (Paulson et al., 2013). PERMANOVA analysis was performed using the vegan package and using 9,999 permutations, and comparisons in α-diversity analysis were performed with the Wilcoxon rank-sum test. To identify plausible biomarkers of sample classes, we used linear discriminant analysis effect size (LEfSe) (Segata et al., 2011) on the CSS-transformed abundances. Network inferences were obtained with SPIEC-EASI (SParse InversE Covariance Estimation for Ecological Association Inference) (Kurtz et al., 2015) using the untransformed ASV count table. This method does not imply correlation analysis to construct microbiota networks; nevertheless, a connection between two nodes (i.e., an edge of the network) denotes a significant linear relationship between the abundance of two ASVs (i.e., nodes of the network). The microbiota interaction network was plotted with the igraph (Csardi and Nepusz, 2006) package.

## Results

### Effects of Alcohol Binges on Gut Microbiota Profile

**Figure 1:**
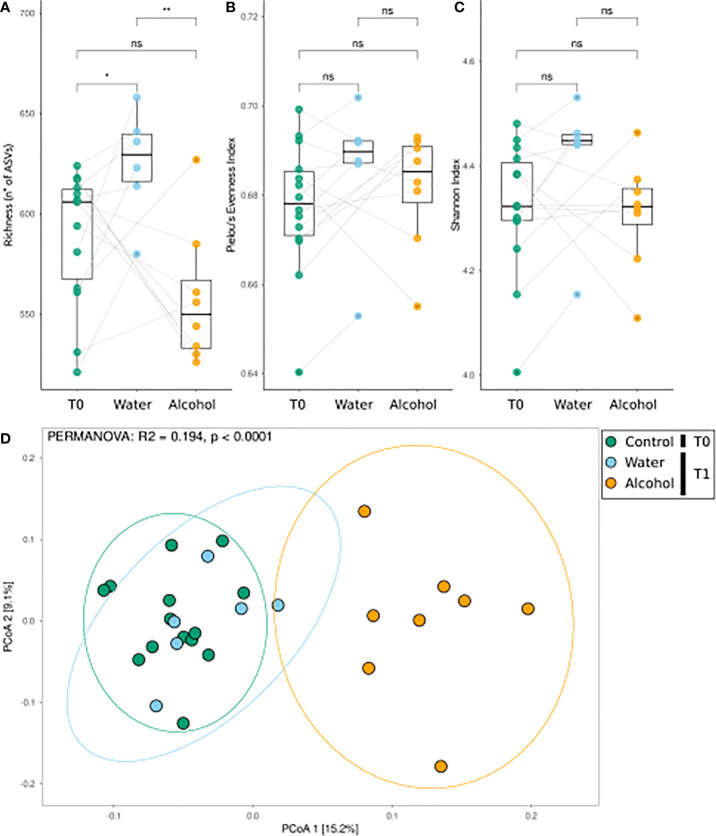
Bacterial community response to alcohol binge drinking. Analyses were performed on the non-OEA-treated sample subset, including the T1 alcohol- and water-administered rats, and the corresponding T0 samples. **(A)** Richness [number of different amplicon sequence variances (ASVs)], **(B)** Pielou’s evenness index (equitability of the distribution of relative abundances among ASVs), and **(C)** Shannon index (diversity of the community) as measures of α-diversity. Differences between groups were tested by the Wilcoxon rank-sum test and reported as notation on the plot (ns, *p*-value > 0.05; *p-value < 0.05; **p-value < 0.01). Dotted lines connect the data from the same animal at the two time points for each treatment. **(D)** β-Diversity analysis with PCoA ordination using the Bray–Curtis distance measure. T0 group, *n* = 14; water group, *n* = 6; alcohol group, *n* = 8. Notation above the plots reports the result of the PERMANOVA test.

We firstly aimed at assessing the effects of alcohol binge drinking on the gut microbiota. To do so, analysis was focused on the subset of animals that did not receive OEA pretreatment, and the effects in the alcohol groups were checked at two time points: before (T0) and after (T1) alcohol binge protocol. Diversity analysis results (reported in **Figure 1**) suggest that alcohol binge drinking had a major effect on the fecal microbiota. Alcohol lowered the richness of the community, which otherwise is significantly increasing from T0 to T1 in the control group (**Figure 1A**), but did not significantly affect the evenness nor the Shannon index (**Figures 1B, C** **)**. In addition, alcohol caused a clearly distinguishable community composition in PCoA ordination analysis, with the alcohol-treated samples forming a distinct cluster along the first ordination axis, significantly different with respect to other samples (**Figure 1D**, see PERMANOVA reported as notation on the ordination graph).

As further confirmation of the strong effect of alcohol binge drinking on the fecal microbiota, we observed a high number of different genera at significantly different relative abundance values among the three sample groups (see **Supplementary Figure 1** for an overall analysis of genera which significantly changed among the three groups, as tested by one-way ANOVA analysis).

**Figure 2:**
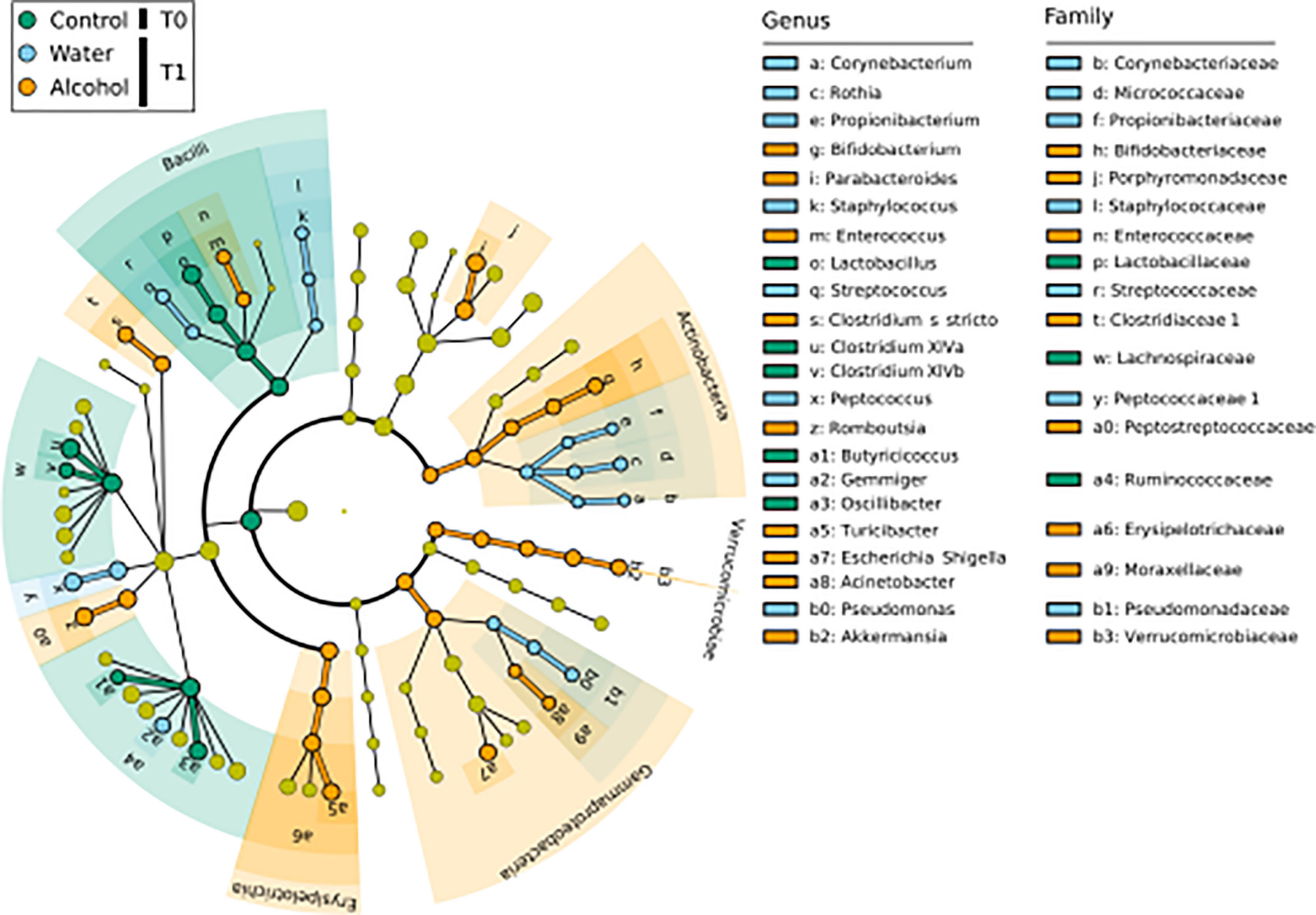
LEfSe biomarker identification for the comparison of genera relative abundance between the T1 ethanol-administered rats, the T1 water-administered rats, and the corresponding T0 samples. Significant biomarker taxon for each group was obtained with a Kruskal–Wallis test among classes (*α* = 0.05], and only biomarkers with a logarithmic linear discriminant analysis score higher than 2.0 were retained. T0 group, *n* = 14; water group, *n* = 6; alcohol group, *n* = 8.

LEfSe analysis (**Figure 2**) was used to discover biomarkers associated to each of the sample groups. To identify those most likely linked to a biological effect, we inspected their relative abundance (i.e., biomarkers with very low median relative abundance in a class are less indicative of a biological effect connected to that class) as well as their frequency distribution (i.e., those markers found in all samples of a class can be considered more likely connected to a biological effect, than those found in only few samples of a class). By doing so, we identify *Clostridium sensu stricto*, *Romboutsia*, *Turicibacter*, *Bifidobacterium*, *Parabacteroides*, and *Enterococcus* as valuable markers for the T1 alcohol group (**Supplementary Figure 2**), and these were found to have high relative abundance levels in ethanol-administrated rats. Conversely, *Lactobacillus*, *Clostridium* XIVa, *Clostridium* XIVb, and *Oscillibacter* were found as markers of T0 samples and displayed a significantly decreasing trend in relative abundance from T0 to T1 (**Supplementary Figure 1**). As such, they could be considered as markers of the normal weaning process. However, given that their decrease in relative abundance was more evident in ethanol-administrated rats, we can attribute it to a specific effect of ethanol on these genera.

### Effects of OEA Treatment on Gut Microbiota Profile

**Figure 3:**
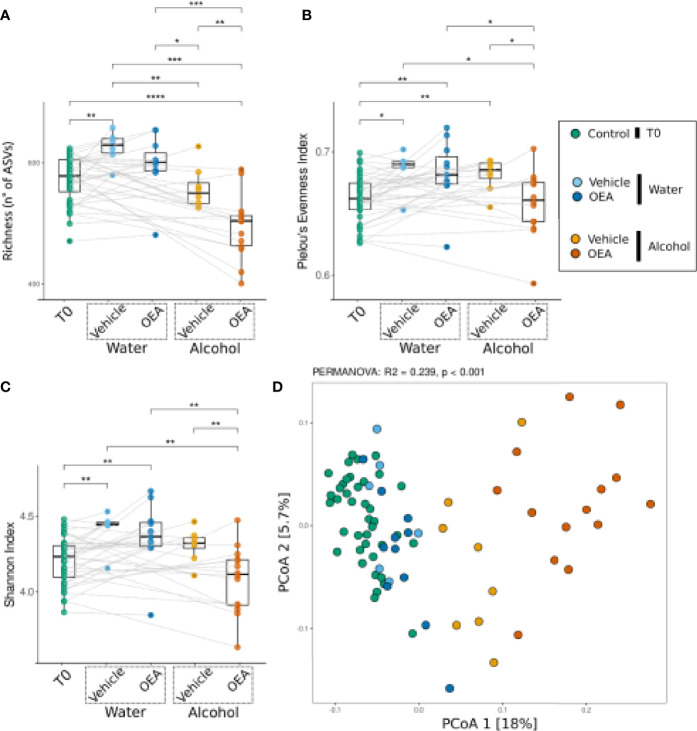
Bacterial community response [α-diversity: **(A–C)**; β-diversity: **(D)**] to alcohol binge drinking and oleoylethanolamide (OEA) treatment. **(A)** Richness (number of different ASVs), **(B)** Pielou’s evenness index (equitability of the distribution of relative abundances among ASVs), and **(C)** Shannon index (diversity of the community) as measures of α-diversity. Differences between groups were tested by Wilcoxon rank-sum test and reported as notation on the plot (*p-value < 0.05; **p-value < 0.01, ***p-value < 0.001, ****p-value < 0.0001). Dotted lines connect the data from the same animal at the two time points for each treatment. **(D)** β-Diversity analysis with PCoA ordination among all samples, using the Bray–Curtis distance measure. Notation above the plots reports the result of the PERMANOVA test. T0 group, *n* = 40; water + vehicle group, *n* = 6; water + OEA group, *n* = 11; alcohol + vehicle group, *n* = 8; alcohol + OEA group, *n* = 14. Notation above the plots reports the result of the PERMANOVA test. Dotted lines connect the data from the same animal at the two time points for each treatment.

After assessing the overall effects of alcohol binge drinking on fecal microbiota, we aimed at analyzing the specific effect of OEA administration per se and in the context of alcohol binge drinking. **Figure 3** reports microbiota α- and β-diversity analysis on all sample sets. Higher richness values were observed in the groups of rats receiving water and vehicle, showing no significant difference with respect to the groups of rats receiving water and OEA, but with a significant difference with respect to samples collected at T0 and to the alcohol-receiving rats (both vehicle- and OEA-treated ones). Alternatively, the lowest richness values were observed in the group of rats receiving alcohol and OEA (**Figure 3A**), with a statistically significant difference *versus* all the other sample groups. The evenness and Shannon indices showed similar trends as both were increased from T0 to T1 except for the group of rats receiving alcohol and OEA, which showed significantly lower values with respect to all other T1 samples and, interestingly, similar to those observed in T0 samples (untreated animals) (**Figures 3B, C** **)**.

β-Diversity analysis (**Figure 3D**) indicated that the community was significantly different in the groups, as confirmed by PERMANOVA results (see notation above the ordination plot in **Figure 3D**). As previously observed, water samples overlap with T0 samples (pairwise PERMANOVA between T0 and water samples were significant but showed low *R* ^2^ values; T0 *vs*. water + vehicle: *R* ^2^ = 0.055, *p*-value: 0.001; T0 *vs*. water + OEA: *R* ^2^ = 0.062, *p*-value: 0.001), showing a separation along the second ordination axis between the vehicle- and OEA-treated groups (pairwise PERMANOVA: water + vehicle *vs*. water + OEA: *R* ^2^ = 0.098, *p*-value: 0.024). Regarding alcohol-treated samples (**Figure 3D**), they are clearly separated from the others along the first ordination axis (representing 18% of total data variance), also showing a difference between OEA and vehicle treatment (pairwise PERMANOVA: alcohol + vehicle *vs*. alcohol + OEA: *R* ^2^ = 0.142, *p*-value: 0.001).

**Figure 4:**
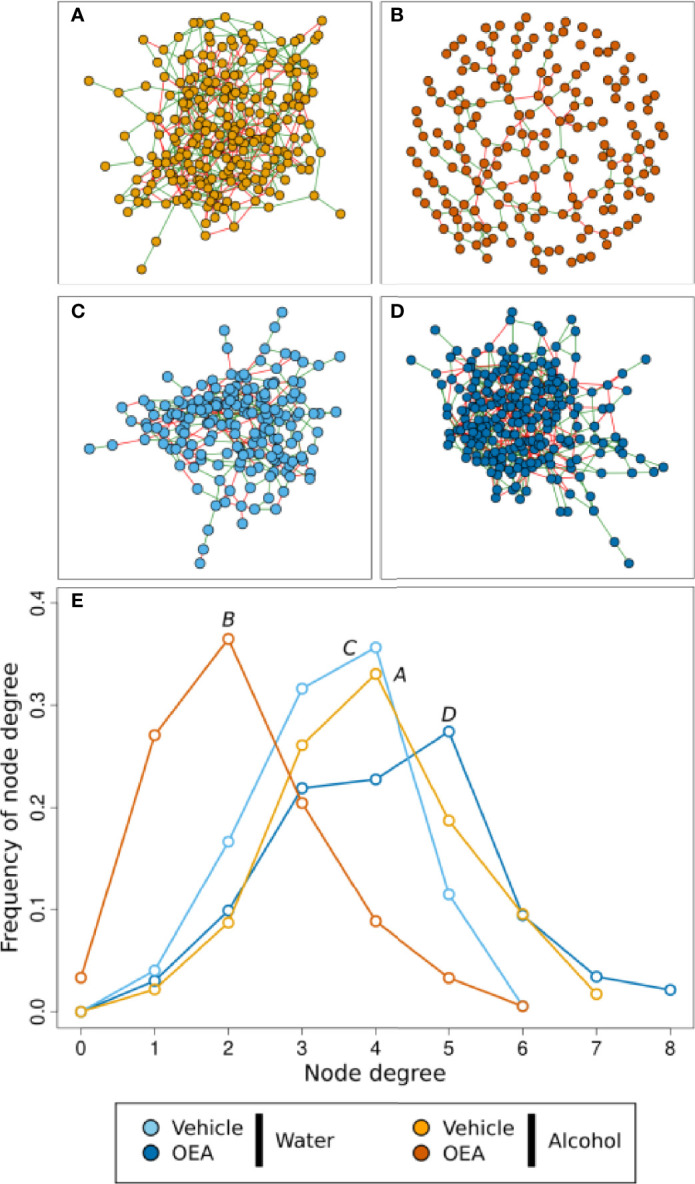
Microbiota association network on the T1 sample subset. **(A–D)** Graphic representation of inferred networks for each group, as indicated by the color of nodes. The graphical representation of the association network is composed of nodes (i.e., circles) connected by edges (i.e., the segments between nodes). Each node represents an ASV of the community, while each edge represents the presence of a significant association between two ASVs. There is a positive association when edges are green, while there is a negative association when edges are red. **(E)** Frequency distribution of the node degree statistic of the four inferred networks. In this representation, we report the frequencies (i.e., % of total) of nodes in the network for each value of node degree (i.e., a statistic representing the number of nodes connected to each node). Each network is represented as a different line, color coded as reported in the legend. Water + vehicle group, *n* = 6; water + OEA group, *n* = 11; alcohol + vehicle group, *n* = 8; alcohol + OEA group, *n* = 14.

To further assess the effects of alcohol binge drinking and the effect of OEA administration in the gut microbiota, we used network analysis to evaluate if the complex interactions between ASVs in each group of samples were different (**Figure 4** and **Table 1**). Overall, the microbial networks in the vehicle-treated samples were most similar (**Figures 4A, C, E** **)**. In both water and alcohol groups, the higher frequency of node degree was 4 and both networks were made of a single connected component (**Figure 4E**, **Table 1**), with the alcohol network resulting as slightly more complex, displaying a higher number of nodes and edges. On the other hand, OEA deeply affected the microbial network structure both in the water and in the alcohol groups, in an opposite direction. The microbial network in the OEA + water group (**Figures 4D, E**, **Table 1**) was the most complex, even in respect to the vehicle networks, with more relations between ASVs (the value of node degree with higher frequency was 5) and with the highest overall number of nodes (ASVs) and edges (significant relations). Notwithstanding, in the alcohol group, OEA administration (**Figures 4B, E**, **Table 1**) had a different effect on the microbial community, leading also to its fragmentation (see the number of components in **Table 1**). In brief, in this network, the value of node degree with higher frequency was 2 and a substantially higher frequency of nodes with node degree equal to 1 or 0 was observed, with respect to the other networks. This means that, in the alcohol + OEA network, 63% of the nodes are linked with only one or two other nodes, whereas 3% of the nodes are not related with any other nodes (unconnected nodes).

**Table 1:** Properties of the networks reported in **Figure 4**.

Network	Total nodes	Total edges	Positive edges (%)	Negative edges (%)	Components	Shortest avg. path length
**Alcohol + OEA**	175	196	146 (75%)	50 (25%)	7	9.06
**Water + OEA**	233	483	278 (57%)	205 (43%)	1	4.35
**Alcohol + vehicle**	230	452	285 (63%)	167 (37%)	1	4.56
**Water + vehicle**	174	292	174 (59%)	118 (41%)	1	5.08

These results suggest that OEA supplementation has a significant different outcome with respect to bacterial community structure and complexity, when combined either with water or alcohol administration. The 10 most abundant genera for each OEA-receiving sample group are reported in **Table 2**. Samples are ordered based on magnitude of the difference in relative abundance between the water and the alcohol sample groups, with one-way ANOVA to test for statistically significant differences. The genera *Bacteroides*, *Escherichia*/*Shigella*, and *Parabacteroides* were significantly more abundant (with a difference higher than 5%) in the alcohol group, while *Clostridium* XIVa, *Lactobacillus*, and *Clostridium* IV genera were significantly more abundant (with a difference higher than 5%) in the water group.

**Table 2:** The 10 most differentiating genera in OEA-supplemented samples (water + OEA and alcohol + OEA).

Bacterial genus	Water + OEAMean % (SD)	Alcohol + OEAMean % (SD)	*p*-value (ANOVA)
***Bacteroides***	26.5 (7.94)	40.3 (9.77)	*
***Escherichia*/*Shigella***	0.12 (0.08)	7.59 (6.88)	*
***Parabacteroides***	7.74 (2.97)	14.3 (5.6)	*
***Peptococcus***	2.00 (1.41)	2.72 (1.32)	ns
***Roseburia***	1.19 (1.02)	1.84 (1.63)	ns
***Flavonifractor***	2.12 (1.74)	2.11 (2.63)	ns
***Alistipes***	6.08 (1.86)	5.99 (3.11)	ns
***Rombutsia***	2.51 (1.34)	1.88 (1.56)	ns
***Ruminococcus***	2.40 (1.93)	1.19 (1.06)	ns
***Oscillibacter***	3.81 (0.978)	2.06 (1.70)	*
***Lachnospiracea inc. Sedis***	4.15 (3.98)	0.37 (0.37)	*
***Clostridium* IV**	6.06 (4.31)	0.77 (0.98)	*
***Lactobacillus***	10.2 (4.47)	0.62 (0.59)	*
***Clostridium* XIVa**	18.5 (7.97)	7.67 (6.89)	*

One-way ANOVA: *p < 0.05. ns > 0.05.

**Figure 5:**
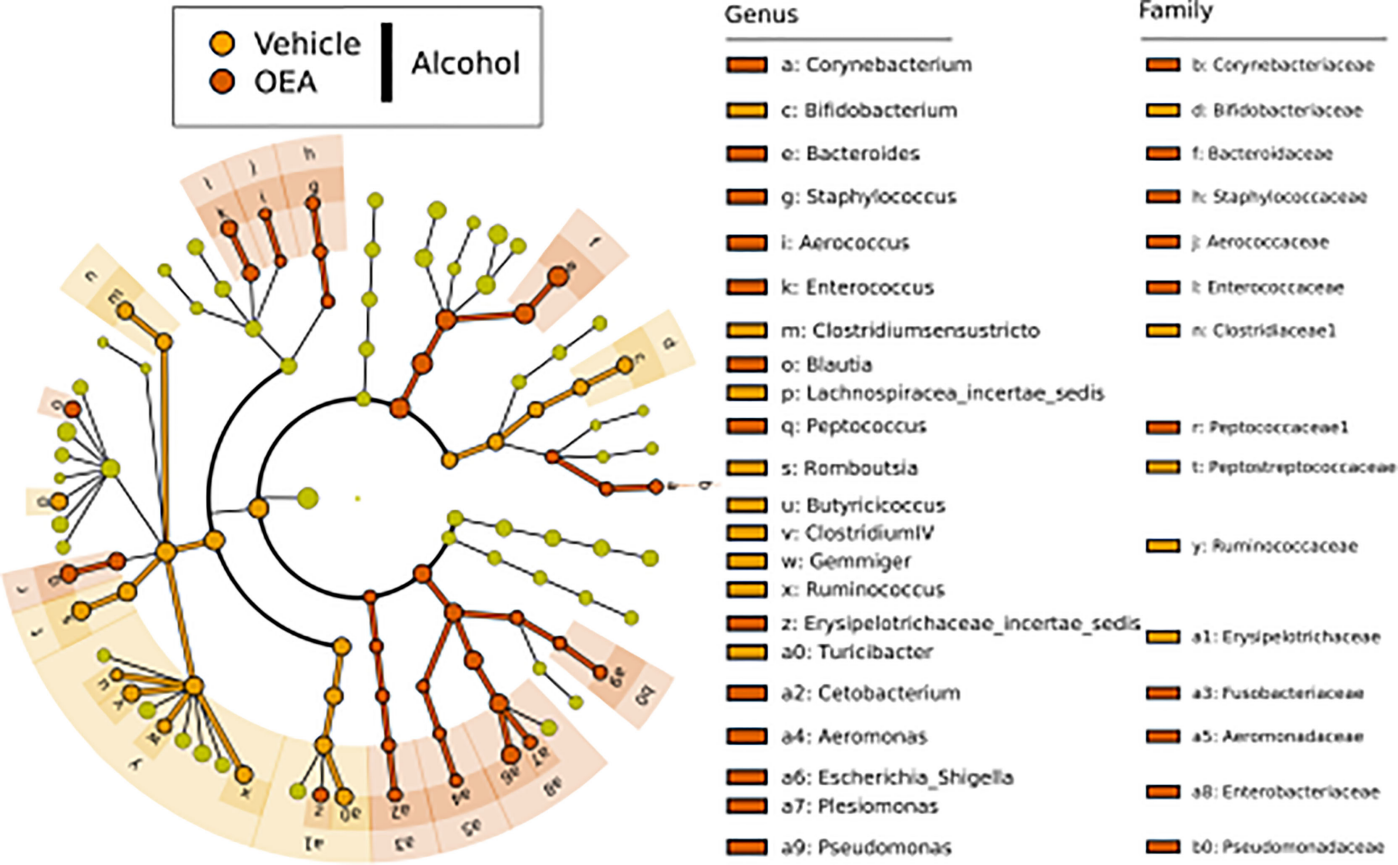
LEfSe biomarker identification for the comparison of genera relative abundance between the OEA-supplemented *vs*. the non-OEA-supplemented group of alcohol-intoxicated rats. Significant biomarker taxon for each group was obtained with a Kruskal–Wallis test among classes (*α* = 0.05), and only biomarkers with a logarithmic linear discriminant analysis score higher than 2.0 were retained. Alcohol + vehicle group, *n* = 8; alcohol + OEA group, *n* = 14.

We next evaluated the effect of alcohol binge drinking and OEA pretreatment on the community composition at the genus level by identifying taxa found at significant different abundance, using LEfSe analysis. As shown in **Figure 5**, comparing alcohol-receiving samples with either OEA or vehicle pretreatment, numerous different taxa showed significantly different relative abundance in the two sample classes. Among them, members of the Firmicutes phylum and, in particular, four genera (*Butyricicoccus*, *Clostridium* IV, *Gemmiger*, and *Ruminococcus*) of the *Ruminococcaceae* family, as well as the genus *Bifidobacterium* of the Actinobacteria phylum, were more abundant in the vehicle group. The OEA-treated samples, conversely, had higher relative abundance of taxa among the Proteobacteria (i.e., *Escherichia*/*Shigella* and *Pseudomonas*) and Bacteroidetes (genus *Bacteroides*) phyla. At the genus levels, *Corynebacterium*, *Staphylococcus*, *Aerococcus*, *Enterococcus*, *Blautia*, and *Peptococcus* were more abundant in the OEA pretreatment group, whereas the genera *Bifidobacterium*, *Clostridium sensu stricto*, and *Turicibacter* were more abundant in the non-OEA pretreatment sample groups.

### Effects of OEA Route of Administration (i.p. *vs*. Oral) on Gut Microbiota Profile

**Figure 6:**
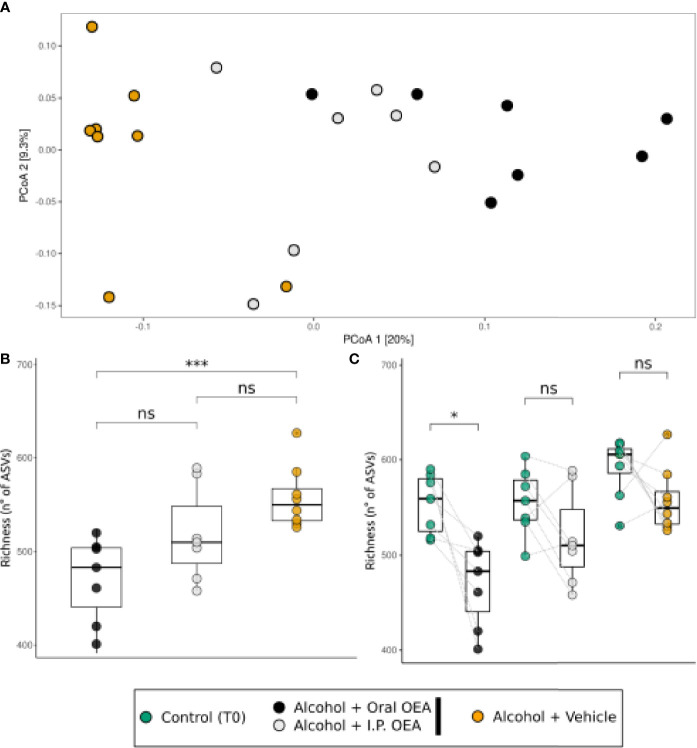
Bacterial community response to different routes of administration of OEA in alcohol-intoxicated rats. **(A)** β-Diversity analysis with PCoA ordination among the oral and i.p. OEA administration route, in comparison with the non-OEA-receiving rats. Ordination was constructed using the Bray–Curtis distance measure. **(B)** Richness (number of different ASVs) in the three sample groups. **(C)** Richness (number of different ASVs) in the three sample groups, in comparison with their respective T0 samples. Differences between groups were tested by Wilcoxon rank-sum test [paired test in panel **(C)**] and the resulting *p*-value was reported as notation on the plot (ns, *p*-value > 0.05; *p-value < 0.05; ***p-value < 0.001). Dotted lines connect the data from the same animal at the two time points for each treatment. T0 group, *n* = 22; alcohol + vehicle group, *n* = 8; alcohol + oral OEA group, *n* = 7; alcohol + i.p. OEA group, *n* = 7.

Having assessed the general effect of OEA on gut microbiota, we finally aimed to discern whether different routes of OEA administration (oral *vs*. i.p.) had a differential impact on gut microbiota in the context of alcohol binge drinking. **Figure 6** reports the diversity analysis on the alcohol-administrated rats receiving oral or i.p. OEA, which are compared with non-OEA alcohol-receiving rats in T1 and with the respective control rats in T0. The separation of samples in the PCoA ordination analysis (**Figure 6A**) indicated that oral and i.p. OEA administration in alcohol-intoxicated animals had a different effect on the structure of microbiota with respect to the vehicle group (ANOSIM test; *R* ^2^ = 0.50; *p* = 1.00e^−4^). This was then confirmed by the pairwise PERMANOVA, which indicated that all the three groups of samples had a significantly different microbial community between each other. The highest differences were found between the vehicle and the oral OEA (*R* ^2^ = 0.2322; *p* = 0.0006) or the i.p. OEA (*R* ^2^ = 0.1423; *p* = 0.0021). The difference between the two administration routes was significant (*R* ^2^ = 0.1367; *p* = 0.0042), which suggests that the OEA administration route may have a fundamental role in shaping the microbiota in alcohol-treated rats.

A similar finding can be observed also for the α-diversity measure of ASV richness (**Figure 6B**). The richness index appears to decrease in the i.p. and oral OEA in comparison with the vehicle group, but the only significant difference was found with respect to the oral OEA. Thus, oral OEA is also the only one showing a significant decrease in richness from T0 to T1 (**Figure 6C**).

**Figure 7:**
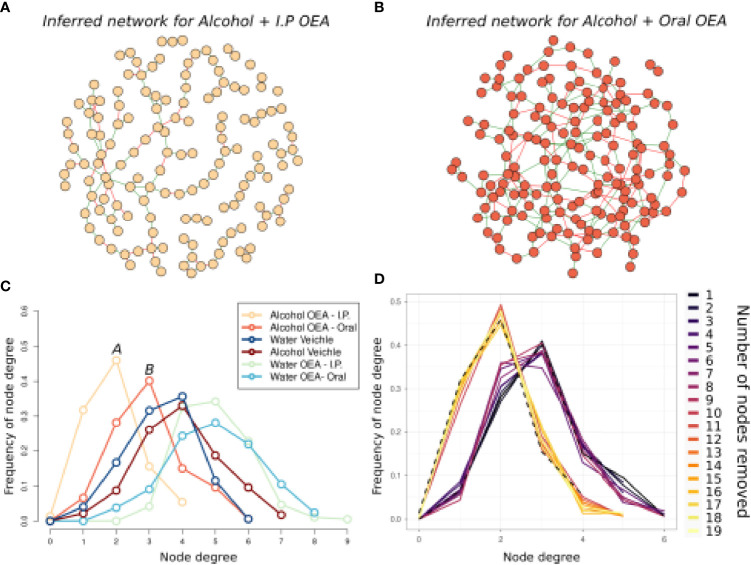
Microbiota association network on the alcohol-intoxicated samples, comparing the oral and i.p. administration routes of OEA. Graphic representation of inferred networks for the i.p. OEA **(A)** and oral OEA **(B)**-receiving rat groups, as indicated by the color of nodes and title. The graphical representation of the association network is composed of nodes (i.e., circles) connected by edges (i.e., the segments between nodes). Each node represents an ASV of the community, while each edge represents the presence of a significant association between two ASVs; there is a positive association when edges are green, while there is a negative association when edges are red. **(C)** Frequency distribution of the node degree statistic of all the inferred networks. In this representation, we report the frequencies (i.e., % of total) of nodes in the network for each value of node degree (i.e., a statistic representing the number of nodes connected to each node). Each network is represented as a different line, color coded as reported in the legend. **(D)** Frequency distribution of the node degree statistic in the oral OEA network, during the “attack” on node betweenness. This is graphically represented as in **(C)**, but in this case, a single line for each iteration of the test is represented, and they all represent the oral OEA network from which a node is removed every iteration. Dotted lines represent i) the starting frequency distribution of the “undisturbed” oral OEA network (on the right) and ii) the frequency distribution of the i.p. OEA network. Each line represents the frequency distribution of node degree of a different network, reobtained after removal of one node (and recalculation) of the previous network. After the removal of 11 nodes, the frequency distribution of node degree in the oral OEA community resembles well the frequency distribution of the node degree in the i.p. OEA community. Alcohol + oral OEA group, *n* = 7; alcohol + i.p. OEA group, *n* = 7.

We further assessed the different effects of oral and i.p. OEA in the alcohol-administered group by evaluating microbiota interactions using network analysis (**Figure 7**). The microbial network induced by i.p. OEA pretreatment (**Figure 7A**) is distinguishable from the one induced by oral OEA pretreatment (**Figure 7B**). The i.p. OEA network was sparser, with roughly 19 nodes less than the oral OEA one, but with almost half the edges (142 *vs*. 246) (**Table 3**). Consequently, the network has a high number of non-interconnected node modules, composed of 13 different components and a frequency distribution of node degree statistic dramatically skewed toward low values (**Figure 7C** and **Table 3**). Interestingly, the same effect was not found for the sample group with water administration (**Figure 7C**), in which there were little differences between i.p. and oral OEA administration. This difference is likely not linked to general ASV richness reduction (i.e., less total ASVs in the community would have less interactions and less nodes); as in **Figure 6**, we showed an opposite trend (higher richness in i.p. OEA with respect to oral OEA with no significant differences).

**Table 3:** Properties of the networks reported in **Figure 7**.

Networks	Total nodes	Total edges	Positive edges	Negative edges	Components	Shortest avg. path length
**OEA i.p.**	148	142	93 (65%)	49 (35%)	13	10.31
**OEA oral**	167	246	148 (60%)	98 (40%)	2	5.96

To elucidate possible mechanisms, we simulated the changes between the i.p. and oral networks by gradually removing 19 ASVs from the data (i.e., the difference in total nodes between the two networks) of the oral OEA samples, recalculating the network, and measuring the frequency distribution of node degree statistic (**Figure 7D** and **Supplementary Figure 3**). We simulated two mechanisms of community reassembly following i.p. OEA administration: a random loss of ASVs (**Supplementary Figure 3**) and a “targeted” loss of ASVs in decreasing order of node *betweenness* (i.e., in decreasing importance for the network structure). We were able to reproduce the same frequency distribution of node degree observed for the i.p. network, by the removal of 11 ASVs in decreasing *betweenness* order in the community of the oral sample, while we did not always reproduce the same distribution when nodes were randomly removed (in 20 repeated simulations). These results indicate that the difference between the community in the oral and i.p. sample groups seems to be at the single ASV level, likely originating from the loss of some specific and highly interacting ASVs and not from random ASV loss.

## Discussion

In this paper, we describe the changes that alcohol binge drinking promotes in the gut microbiota during a 4-day protocol *versus* control (water) animals and the influence of i.p. and oral OEA administration in healthy conditions and in alcohol-induced dysbiosis.

Our results indicate that alcohol binge drinking reduces the bacterial α-diversity (richness of ASVs) and creates a clearly distinguishable community in ordination analysis (different β-diversity), with main differences observed from T0 (no treatment) to T1 (last day of binge) in the binge drinking group only. Also, alcohol binge drinking promotes some bacterial community compositional differences at the genus and family levels. Remarkably, alcohol binge drinking promotes a significant decrease in *Lactobacillus*, *Clostridium*, and *Oscillibacter* genera and the increment of *Bifidobacterium*, *Turicibacter*, *Parabacteroides*, and *Romboutsia*. An alcohol-induced decrement of *Lactobacillus* was previously reported in animal models using different patterns of alcohol consumption (Yan et al., 2011; Hartmann et al., 2013; Xiao et al., 2018; Kosnicki et al., 2019; Lee et al., 2019; Lamas-paz et al., 2020). Regarding α- and β-diversity, other studies using different alcohol models have obtained diverging results, some showing alcohol-induced reduction in α-diversity and/or changes in β-diversity (Mutlu et al., 2009; Bull-Otterson et al., 2013; Lee et al., 2019; Samuelson et al., 2019; Zallar et al., 2019) and others without significant changes in α- or β-diversity (Neyrinck et al., 2016; Reyes et al., 2020). At the time of the present study, the specific impact of alcohol on gut microbiota when consumed in a binge drinking pattern remained elusive. A recent study has shown that mice under a drinking in the dark (DID) alcohol binge model neither show changes in α-diversity (Shannon, Simpson, and Chao1 indices) or β-diversity nor show significant changes in bacteria community composition at the phylum or family level (Reyes et al., 2020). As mentioned before, in the present study, we observed alterations in both α- and β-diversity with a 4-day alcohol binge drinking protocol in rats. Differences in the protocol and/or the animal species may account for these discrepancies.

It is very interesting to note that changes in bacterial composition have been observed in different neurological disorders, although their causal roles remain elusive, and observations often remain limited to a correlational level. For example, a reduced abundance of *Bifidobacterium* and an increment of *Clostridia* were observed in autism, while a reduced abundance of *Bifidobacterium* and *Lactobacillus* was observed in depression (reviewed in Cryan et al., 2019). Additionally, the use of psychobiotics (probiotics that when consumed promote a beneficial effect on mental health; Dinan et al., 2013) has been shown to improve concentration and decrease anxiety and depression symptomatology both in preclinical and clinical studies (reviewed in Scriven et al., 2018). The specific consequences of alcohol binge-induced dysbiosis presented in this study are at present unknown as our results are descriptive and, thus, do not allow for mechanistic speculation about possible implications of these bacterial changes in a healthy status.

Previous experiments in Orio’s lab indicated that OEA pretreatment before each alcohol gavage has protective actions against multiple alcohol-induced effects, such as neuroinflammation, leaky gut, anhedonia, or depressive-like behavior in abstinence (Sayd et al., 2015; Antón et al., 2017; Antón et al., 2018; Orio et al., 2019; Orio, 2020). The actions of OEA were particularly interesting in the gut, since this biolipid was able to strongly reduce gut inflammation and colonic TJ disruption, providing protection against the bacteria translocation and also reducing alcohol-induced alterations in the immune system. Here, we aimed to fully characterize the actions of OEA in the gut by studying its impact on microbiota in the context of binge drinking. We hypothesized that some of the protective actions of OEA in the gut–brain axis could be mediated by amelioration of alcohol-induced gut dysbiosis. Results obtained in the present study are intriguing, since the effects of OEA significantly differ when this biolipid is administered in control or in alcohol-treated animals. Surprisingly, pretreatment with OEA in the alcohol-administered rats decreased the α-diversity by a reduction in evenness and Shannon indices, as well as changed β-diversity. Additionally, OEA pretreatment induced major changes in *Bacteroides*, *Clostridium*, *Escherichia*/*Shigella*, and *Rombustia* genera and promoted a fragmentation of the microbial network in the alcohol group. The latter evidence can be interpreted as indicative of a detrimental effect of the administration of OEA in conjunction with alcohol treatment. In fact, a more complex interaction network (i.e., with a frequency distribution skewed toward a higher degree) is indicative of a more diverse and more functional community in the intestinal environment, whereas shorter average path length values can be interpreted as increasing the speed at which the network responds to perturbations (Faust and Raes, 2012). It is to note that the α-diversity indices in the combined OEA and alcohol group are more similar to untreated rats (T0) than any other groups.

Nevertheless, the effect of OEA in physiological conditions (no administration of alcohol) appears to be different, showing no changes in α-diversity and only a slight change in β-diversity. At the community composition level, OEA increased *Bacteroides* and reduced *Lactobacillus*, among other genera. These results agree with our previous results using 10 mg/kg i.p. OEA during 11 consecutive days (Di Paola et al., 2018). In the mentioned paper, OEA increased Bacteroidetes phylum (*Bacteroides*) and decreased Firmicutes (*Lactobacillus*) and changed β-diversity; OEA, though, did not induce significant changes in α-diversity (Di Paola et al., 2018). As commented in the discussion of the cited paper, these results suggest that OEA treatment promotes a shift in gut microbiota toward a “lean-like phenotype”, mimicking the effects of a low-fat and high-polysaccharide/fiber-rich diet. Intriguingly, contrary to what was previously observed, OEA treatment without alcohol administration led to opposite changes in the microbial interaction network, which could be interpreted as positive. OEA treatment in this case increased the node degree value with higher frequency, skewing its distribution to the right, and increased total nodes and edges in comparison with the control group.

These results suggest that OEA may ameliorate the balance of gut bacteria in physiological conditions, but not under alcohol intoxications. Although unexpected, those results may also indicate that the beneficial effects of OEA pretreatment in the context of alcohol binge drinking are not mediated by changes in gut bacterial composition. Whether bacteria change their activity in different settings (with and without alcohol or its products, in conjunction to OEA pretreatment or not) is for us at present unknown. Other mechanisms, such as reduction of colonic inflammation, tight junction disruption and bacterial translocation, regulation of the immune system, antioxidant actions, and/or reduction of neuroinflammation, have been proposed to explain the protective actions of OEA in alcohol binge (Orio et al., 2019; Orio, 2020; Rodriguez-Gonzalez and Orio, 2020).

It is intriguing that the water group varies from T0 to T1. The reasons for the α-diversity changes from T0 to T1 in this control group are not fully understood. It has been suggested elsewhere that they could reflect the normal aging of animals (Flemer et al., 2017), although our experiment was too short to question this hypothesis. It is to note that the present experiment was designed to depict the specific effects of OEA in gut microbiota, and because of that, all animals in T1, including the water group, were injected with vehicle or with OEA. This means that, with this experimental design, we cannot discard the effect of the vehicle in the water-administered animals. Indeed, significant effects of vehicle in gut microbiota were found in some studies using long-lasting oral treatments, although the effects were considered detrimental. For example, continued oral administration of 1% Tween 80 or carboxymethylcellulose, as dietary emulsifiers, induced microbiota alterations and changed β-diversity when given in drinking water for 12 weeks (Chassaing et al., 2015; Holder et al., 2019). Our experiment was designed to question the specific effects of OEA in gut microbiota, and although the time of treatments was shorter, our experimental design did not allow to discard or confirm any vehicle effect.

Another interesting finding of this study is that the route of administration of OEA may affect differently the gut microbiota. Results indicate that oral OEA, but not i.p. OEA, significantly reduced richness in comparison with the vehicle + alcohol group, as well as oral and i.p. OEA differently changed bacterial community structure with respect to the mentioned group. Although the biological significance of many of the bacterial changes observed in this study is still unknown, we observed that i.p. OEA administration resulted in a more fragmented network with more non-interconnected nodes, suggesting a more detrimental effect on the microbial network with respect to oral administration. This difference in network community may be due to the loss of some specific highly interacting ASVs. It is to note that we previously observed differences between oral and i.p. OEA pretreatment in the context of alcohol binge drinking using the same animal model. As mentioned before, we showed that i.p. OEA was able to prevent the alcohol binge drinking-induced neuroinflammation in the frontal cortex, colonic inflammation, and decrement of gut tight junction proteins. However, oral OEA resulted to be more effective in preventing alcohol-induced gut bacteria translocation to MLNs and spleen and the passage of the high inflammatory component, LPS, to the systemic circulation (Antón et al., 2018).

In conclusion, to the best of our knowledge, this is the first paper studying the effect of alcohol binge drinking on fecal microbiota using this model and exploring the potential protective effect of pretreatment with the biolipid OEA on dysbiosis induced by binge drinking. They are both unexplored fields, and some of the results we have obtained are different from our initial hypothesis, which has caused difficulties in interpreting the results and understanding the biological meaning. Some of these difficulties arise from possible limitations of this study. For example, we did not have the possibility of measuring any biological marker (i.e., proinflammatory cytokines TNF-α or IL-1β in plasma) to correlate with the gut microbiota changes; this would have helped us to better understand the biological meaning of the observed microbial changes. Also, here, we used fecal samples as proxies for colonic microbiota content. Despite differences in the composition between fecal and mucosal communities being increasingly investigated, feces remain the most used sample source for gut microbiota analysis because it is naturally collected and non-invasive and can be obtained repeatedly (Tang et al., 2020). However, we cannot rule out that a similar analysis performed on samples of small intestine could produce different results than those presented here. In fact, it has been shown that chronic alcohol consumption may have a different impact on microbiota composition structure in the colon and in the jejunum (Fan et al., 2019). In conclusion, we carried out observational experiments, and despite the limitations, the observed results set the basis for future research to understand the biological meaning of these alterations.

To sum up, our results suggest that the protective actions of OEA in the context of alcohol abuse are not related to changes in alcohol-induced dysbiosis. Other mechanisms previously identified such as the prevention of leaky gut and anti-inflammatory actions may account for the protective effects.

## Data Availability Statement

Datasets are uploaded in the European Nucleotide Archive (ENA) database with accession number PRJEB44965.

## Ethics Statement

The animal study was reviewed and approved by the Animal Welfare Committee of Complutense University of Madrid (reference: PROEX 420/15).

## Author Contributions

AR-G, MM, and LO were in charge of the experiment design and activity related to alcohol binge drinking in laboratory animals and sample collection. FV, CF, and MBP were in charge of microbiota analyses and statistical measures. AR-G, FV, and LO drafted the manuscript. All authors contributed to the article and approved the submitted version.

## Funding

This work was supported by FEDER (European Union)/Ministerio de Ciencia e Innovación/Agencia Estatal de Investigación (Spain) (grant number: Retos 2018; RTI2018-099535-B-I00 to LO). AR-G was a recipient of a fellowship from Consejería de Educación, Juventud y Deporte (Comunidad de Madrid/Fondo Social Europeo). MBP was supported by JPI Nutricog, grant “Ambrosiac”.

## Conflict of Interest

The authors declare that the research was conducted in the absence of any commercial or financial relationships that could be construed as a potential conflict of interest.

## Publisher’s Note

All claims expressed in this article are solely those of the authors and do not necessarily represent those of their affiliated organizations, or those of the publisher, the editors and the reviewers. Any product that may be evaluated in this article, or claim that may be made by its manufacturer, is not guaranteed or endorsed by the publisher.

## Supplementary Material

Supplementary Figure 1 Comparison of genera relative abundance between the T1 alcohol administered rats, the T1 water administered rats, and the corresponding T0 samples. Only the genera with significant (with p-value < 0.05) difference in one way ANOVA were reported.

Supplementary Figure 2 Best subset of identified microbiota markers, as reported in Figure 2. Histograms report relative abundance distribution among individual samples, grouping samples in the T1 alcohol and T1 water administered rats, and the corresponding T0 rats. The best marker were identified by evaluating their prevalence (i.e. the presence in multiple samples of a class) and relative abundance (i.e. by evaluating mean and median group abundances, reported as solid and dashed lines, respectively).

Supplementary Figure 3 Microbiota association network on the alcohol intoxicated samples, comparing oral and i.p administration route of OEA. Image reports frequency distribution of the node degree statistic in the oral OEA network, during the removal of random nodes. Dotted lines represent i) the starting frequency distribution of the “undisturbed” oral OEA network (on the right,), ii) the frequency distribution of the i.p. OEA network. Each line represents the frequency distribution of node degree of a different network, re-obtained after removal of one node (and recalculation) of the previous network. The procedure was repeated 20 times. As none of the repetition showed a change in the shape of frequency of node degree distribution from the undisturbed network, we conclude that the loss of specific ASVs from the community led to the observed changes in microbial association network between the oral and the i.p. samples community.
