# Supplementary figures and images for: Effects of Alcohol Binge Drinking and Oleoylethanolamide Pretreatment in the Gut Microbiota
Source: Front Cell Infect Microbiol. 2021 Nov 23;11:731910. doi: 10.3389/fcimb.2021.731910 (PMC8651011; doi:10.3389/fcimb.2021.731910)

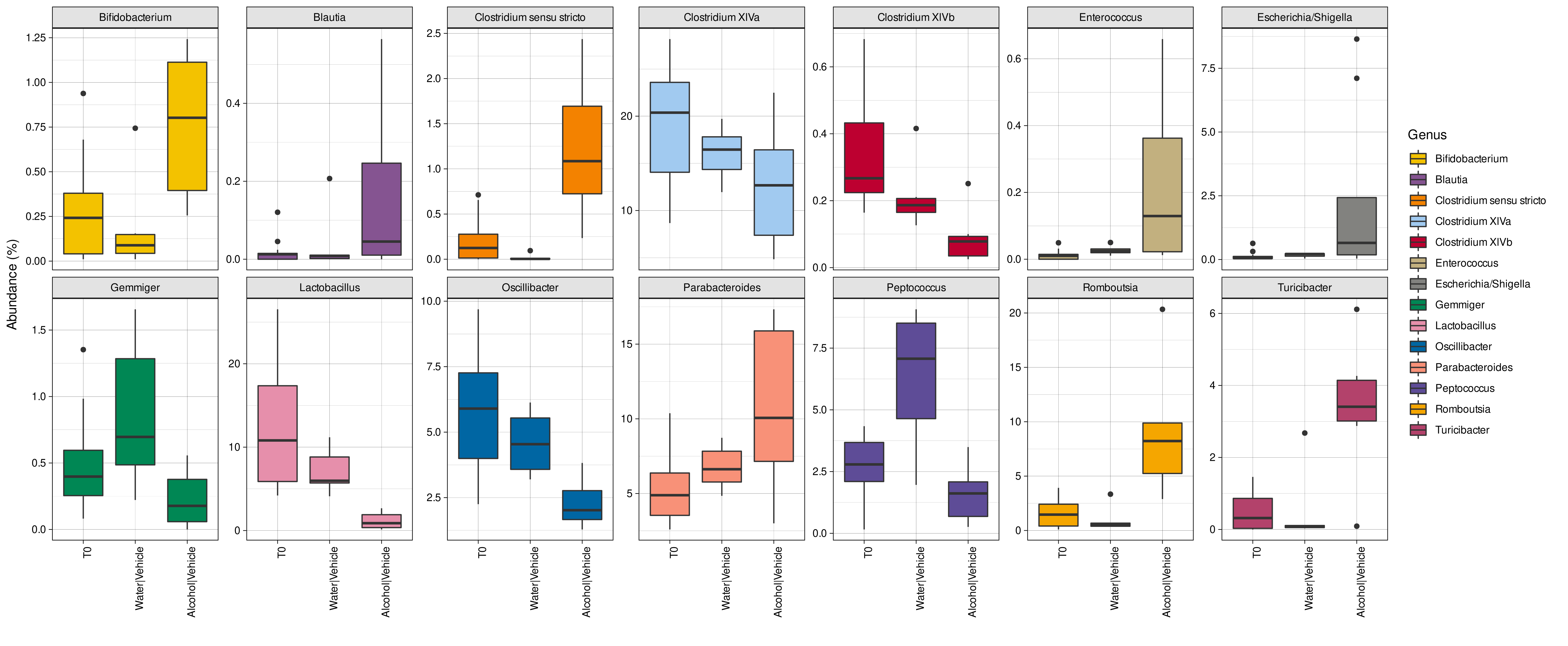

Supplement: Supplementary Figure 1 — Comparison of genera relative abundance between the T1 alcohol administered rats, the T1 water administered rats, and the corresponding T0 samples. Only the genera with significant (with p-value < 0.05) difference in one way ANOVA were reported. [file Image_1.tiff]

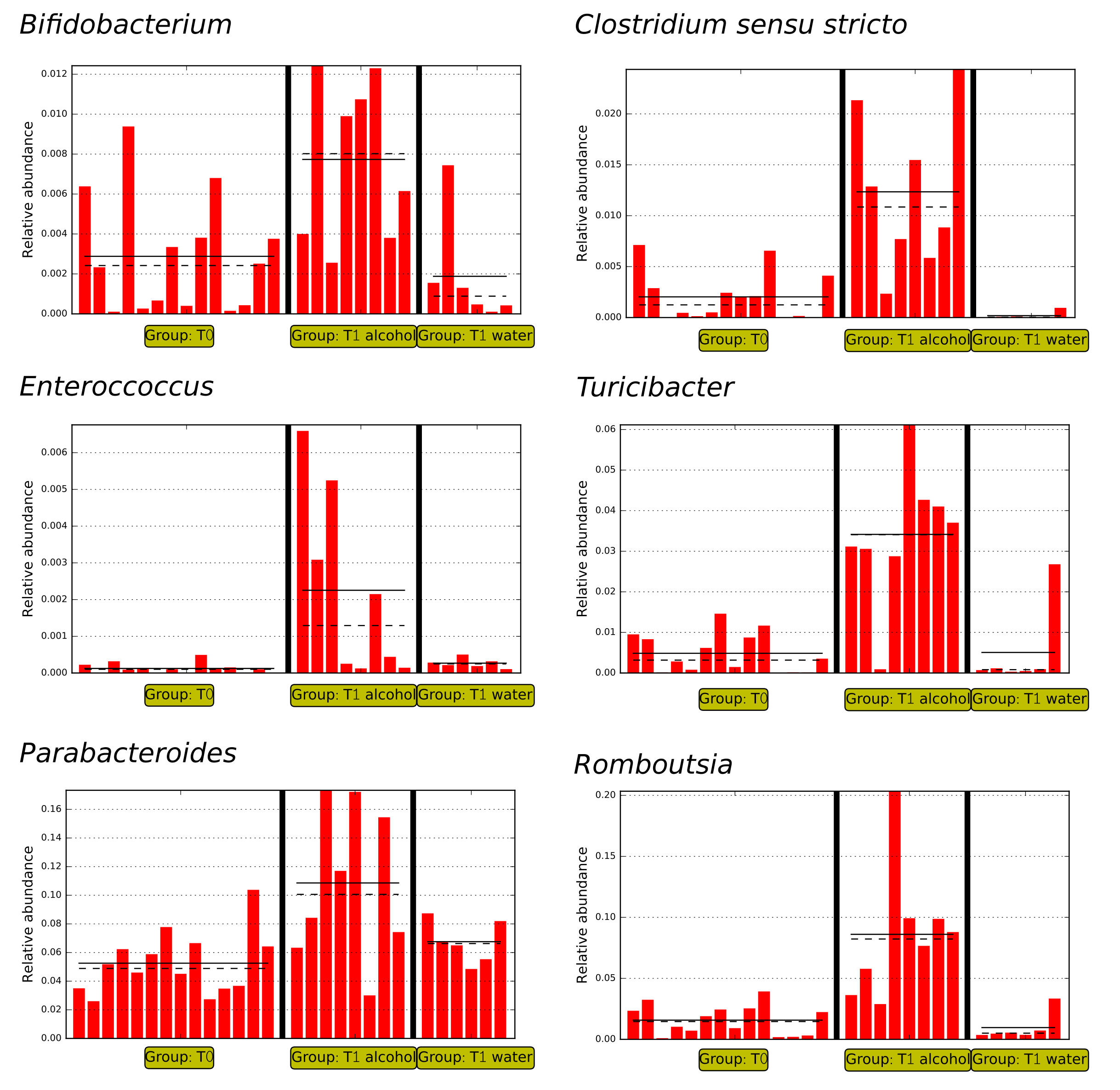

Supplement: Supplementary Figure 2 — Best subset of identified microbiota markers, as reported in Figure 2. Histograms report relative abundance distribution among individual samples, grouping samples in the T1 alcohol and T1 water administered rats, and the corresponding T0 rats. The best marker were identified by evaluating their prevalence (i.e. the presence in multiple samples of a class) and relative abundance (i.e. by evaluating mean and median group abundances, reported as solid and dashed lines, respectively). [file Image_2.tiff]

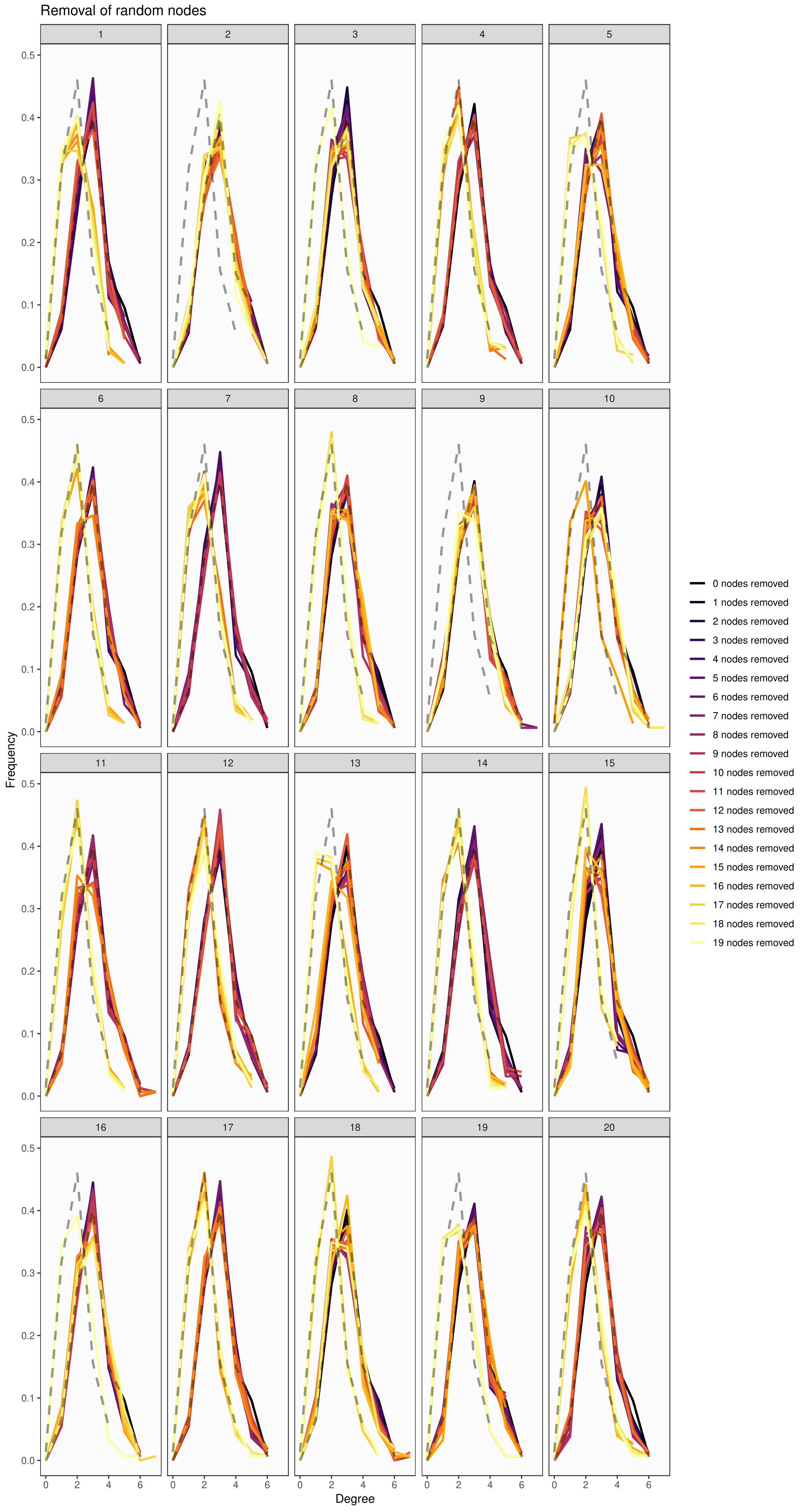

Supplement: Supplementary Figure 3 — Microbiota association network on the alcohol intoxicated samples, comparing oral and i.p administration route of OEA. Image reports frequency distribution of the node degree statistic in the oral OEA network, during the removal of random nodes. Dotted lines represent i) the starting frequency distribution of the “undisturbed” oral OEA network (on the right,), ii) the frequency distribution of the i.p. OEA network. Each line represents the frequency distribution of node degree of a different network, re-obtained after removal of one node (and recalculation) of the previous network. The procedure was repeated 20 times. As none of the repetition showed a change in the shape of frequency of node degree distribution from the undisturbed network, we conclude that the loss of specific ASVs from the community led to the observed changes in microbial association network between the oral and the i.p. samples community. [file Image_3.tiff]
